# An aggregation-removal model for the formation and size determination of post-synaptic scaffold domains

**DOI:** 10.1371/journal.pcbi.1005516

**Published:** 2017-04-24

**Authors:** Jonas Ranft, Leandro G. Almeida, Pamela C. Rodriguez, Antoine Triller, Vincent Hakim

**Affiliations:** 1 Laboratoire Physique Statistique, CNRS, Ecole Normale Supérieure, PSL Research University, Université Pierre et Marie Curie, Paris, France; 2 IBENS, Ecole Normale Supérieure, PSL Research University, CNRS, INSERM, Paris, France; Radboud Universiteit Nijmegen, NETHERLANDS

## Abstract

The formation and stability of synapses are key questions in neuroscience. Post-synaptic domains have been classically conceived as resulting from local insertion and turnover of proteins at the synapse. However, insertion is likely to occur outside the post-synaptic domains and advances in single-molecule imaging have shown that proteins diffuse in the plane of the membrane prior to their accumulation at synapses. We quantitatively investigated this scenario using computer simulations and mathematical analysis, taking for definiteness the specific case of inhibitory synapse components, i.e., the glycine receptor (GlyR) and the associated gephyrin scaffolding protein. The observed domain sizes of scaffold clusters can be explained by a dynamic balance between the aggregation of gephyrin proteins diffusing while bound to GlyR and their turnover at the neuron membrane. We also predict the existence of extrasynaptic clusters with a characteristic size distribution that significantly contribute to the size fluctuations of synaptic domains. New super-resolution data for gephyrin proteins established the existence of extrasynaptic clusters the sizes of which are consistent with the model predictions in a range of model parameters. At a general level, our results highlight aggregation with removal as a non-equilibrium phase separation which produces structures of tunable size.

## Author summary

Synapses mediate information transmission between neurons and are the physical support of memory. It has been realized that synapses are dynamic biological structures. Neurotransmitter receptors diffuse in the neuron membrane and synaptic scaffolding proteins are constantly renewed. We propose a biophysical model that links these different measured quantities for inhibitory synapse components and show how they determine the size of postsynaptic domains. The model predicts that synaptic scaffolds also exist extrasynaptically and that they contribute to fluctuations of synaptic sizes. We confirm by super-resolution microscopy the existence of extrasynaptic scaffolds. Their measured size distribution agrees with the model predictions for specific parameters. The model should be helpful to better understand the dynamics of synapses and their possible levels of regulation.

## Introduction

Synapses mediate transmission of information between neurons and are generally thought to be, at least in part, the support of memory. However, when investigated at the molecular scale, synapses appear as dynamic assemblies, the constituents of which are exchanged on timescales ranging from tens of minutes down to seconds [1]. This raises fundamental questions about the way memory is maintained [2]. On the postsynaptic side, neurotransmitter receptors are mostly inserted in the neuron plasma membrane at non-synaptic loci [3–5]. Single-particle imaging and tracking techniques have shown that they subsequently diffuse in the plasma membrane in and out of synaptic domains (see [1] for a review and references therein). The postsynaptic density (PSD) contains scaffolding proteins, which provide binding sites for the receptors and transiently stabilize them at the PSD. The strength of a synapse is determined by the number of receptors at the PSD at a given moment. This number depends on the number of receptor binding sites provided by the scaffolding proteins [6] as well as on the affinity between receptors and scaffolding proteins [7]. Therefore, the size of the PSD, i.e., the number of scaffold protein binding sites, is a key determinant of synaptic strength.

We have focused our study on inhibitory synapses for which most components have been identified and functionally characterized. The gephyrin scaffolding protein is the central structural component of inhibitory synapses [8]. In most cases, scaffolding proteins, including gephyrin [9, 10], are renewed on timescales of minutes to hours in the synaptic domain (for a review see [11]). The basic oligomeric form of a full-length gephyrin is that of a trimer, mediated via strong interactions of the N-terminal G-domains [12–14]. In eukaryotic cells, gephyrin trimers eventually undergo further oligomerization [15], supposed to underlie postsynaptic clustering in neurons. Gephyrin is present in the cellular cytoplasm but it can also diffuse just below the plasma membrane when bound to receptors [16].

In the PSD, gephyrin proteins form homo-multimeric scaffolds just below the plasma membrane. Precise data have been gathered in the past years about several biophysical parameters governing the dynamics of receptors and scaffolding proteins, as reviewed in [1]. GlyR diffusion constants and concentrations inside and outside inhibitory synapses have been determined (see [17] and references therein). The size distributions of PSD and gephyrin scaffolds have been measured as well [6].

A model for the synapse based on these data and linking them is needed to address the questions of synapse formation, maintenance and dynamics from a precise biophysical viewpoint. Co-expression in fibroblasts of GlyR and gephyrin is sufficient to generate at the plasma membrane clusters of sizes similar to that of the PSD at inhibitory synapses [9]. This finding has provided a motivation to investigate theoretically how receptor diffusion, association of receptors to scaffolds, and the self-aggregation of scaffolding proteins could give rise to the formation of domains of given sizes. Some previous works have started to tackle this question. Reaction-diffusion equations for the above-described processes were proposed, and the authors suggested that a Turing-like instability could underlie the formation of synaptic domains [18, 19]. In another study, only receptors were described and it was alternatively proposed that synaptic receptor clusters could result from a dynamic balance between an incoming flux of diffusing receptors and an efflux from the cluster to the cell cytoplasm mediated by receptor removal [20]. That diffusion, aggregation and removal of molecular components can serve to produce macromolecular domains in a membrane has been previously proposed for the formation and maintenance of lipid rafts and precisely examined in that context [21, 22]. Similar mechanisms have also been shown to control the organization or E-cadherin clusters at cell membranes [23]. It is clear that the lifetime of a cluster of proteins can be many orders of magnitude larger than the lifetime of its constituents, an attractive feature for a memory storing structure [2] which has been previously theoretically examined [24].

Based on these previous works, we have now examined the characteristics of scaffold domains produced by scaffolding proteins (here gephyrin) bound to receptors (here GlyR) diffusing in the plasma membrane. Taking into account the available biophysical data [6, 9, 16] we first deduce that the combined effect of aggregation, diffusion and removal produces typical scaffold domain sizes which are similar to those observed experimentally. Then, particle-based computer simulations and theoretical analyses allow us to comprehensively characterize the cluster sizes and dynamics resulting from these basic processes. In agreement with previous works on aggregation–removal in other contexts [25, 26], we show that aggregation and removal of scaffolding proteins provide a non-equilibrium process at the origin of the distribution of dynamically evolving scaffold domains of different sizes. Furthermore, we predict how the size distribution of these domains depends on biophysical parameters such as the turnover time of scaffolding proteins at the plasma membrane and the dependence of cluster diffusion with size.

The actual size distribution of gephyrin clusters was measured in cultured spinal cord neurons using super-resolution microscopy. By comparing these novel observations with model predictions, we then infer the biophysical parameters that govern the assembly of gephyrin domains according to the proposed model of protein aggregation, diffusion, and turnover.

## Results

### Balance between lateral aggregation and cytoplasmic recycling of scaffold proteins sets scaffold domain size

We set out to assess the combined role of

**Fig 1:**
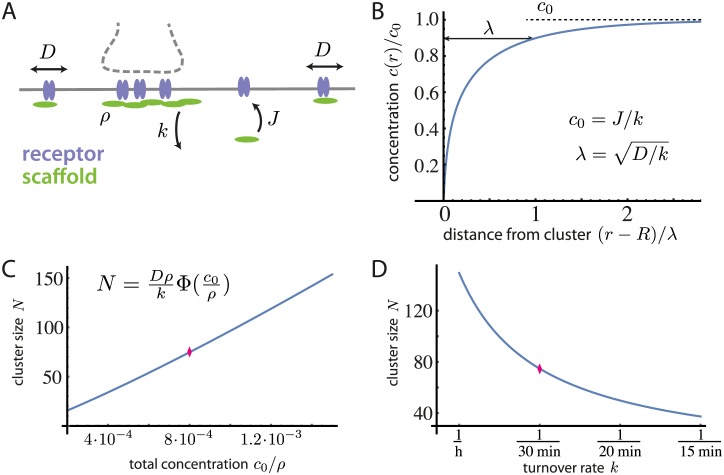
Lateral diffusion and aggregation of scaffold-receptor complexes at the post-synaptic membrane. A: Sketch of the model basic processes: receptors (GlyR, blue) and scaffold “particles” (gephyrin trimers, green) can form complexes; scaffold particles are brought to the cell membrane with a flux *J* and leave it with a rate *k*; sub-membraneous scaffold particles aggregate by homotypic scaffolding protein interactions; scaffold-receptor complexes diffuse laterally along the cell membrane, with a diffusion constant *D*. B: Concentration profile of diffusing scaffold particles around a disc-shaped scaffold domain of radius *R*. Far from the cluster, the concentration of diffusing scaffold particles is uniform and equal to *J*/*k*. The diffusing scaffold particles are depleted in a layer of size $\lambda =\sqrt{D/k}$ close to the cluster boundary. C: Dependence of the stationary domain size *N* on the particle concentration *c*_0_. D: Dependence of *N* on the turnover rate *k*. Reference parameters are taken to be *D* = 0.02 *μ*m^2^/s [16], *k* = 1/(30 min) [9], *c*_0_ = 4/3 *μ*m^−2^ [16, 27]. The concentration of scaffold particles/trimers in a post-synaptic domain is taken to be *ρ* = 5000/3 *μ*m^−2^ [6]. When parameters are varied in B and C, the reference values are marked (red losange).

1. lateral diffusion of bound scaffold-receptor complexes in the membrane,
2. scaffolding protein aggregation, and
3. scaffolding protein removal at the membrane and in scaffolds

by first analyzing a reduced model of scaffolding protein dynamics at the membrane, see Fig 1. In this model, a single scaffold domain is surrounded by diffusing scaffolding “particles” (i.e. gephyrin trimers) bound to receptors. The domain edge acts as an absorbing boundary on the diffusing complexes, thus imposing a concentration gradient which in turn gives rise to an incoming diffusive flux of scaffold proteins.

Because this influx grows at most like the perimeter of the domain, while the protein efflux due to desorption of aggregated scaffolding proteins into the cytoplasm scales with the area of the domain, the balance of both fluxes occurs for a well-defined domain size. The resulting equilibrium size can be calculated analytically (see S1 Text: Single scaffold domain) and depends on biophysical parameters such as the diffusion coefficient of scaffold-receptor complexes in the extrasynaptic membrane *D*_0_, the removal rate of scaffold proteins *k*, the total surface concentration of scaffold proteins at the membrane *c*_0_, and the density of scaffold proteins within the domain *ρ*. Note that *k* is an effective rate that captures any local imbalance of binding and unbinding of scaffold particles from and into the cytoplasm, respectively. Measured in the number *N* of scaffold particles, its “building blocks”, the expression for the domain size reads

$$N=\frac{\rho D_{0}}{k}\Phi \left(\frac{c_{0}}{\rho }\right)\;.$$ (1)

(The explicit form of the function Φ(*x*) is given by Eq. (S5,S7) in S1 Text: Single scaffold domain.) In Fig 1C and 1D, the domain size *N* is shown as a function of *c*_0_ and *k*, respectively.

To check the plausibility of scaffold formation and maintenance by diffusion and removal, we calculate the expected domain size using the above equation and parameter estimates for gephyrin and GlyR from the literature, see Fig 1. Assuming that the smallest occurring gephyrin unit or “building block” are gephyrin trimers [12–14], we obtain an approximate domain size of *N* ≃ 70 trimers, or 210 gephyrin monomers, which is surprisingly close to previously published measurements of gephyrin domain sizes [6]. We therefore conclude that diffusion, aggregation, and turnover may indeed be key mechanisms involved in setting the size of synaptic scaffold domains.

The above analysis explicitly relates the size of post-synaptic gephyrin domains to the measured diffusion constant of GlyRs and the turnover rate of gephyrin in scaffold domains. It is however based on the simple assumption of a single circular gephyrin domain surrounded by diffusing GlyR-gephyrin trimer complexes. To further investigate the proposed scenario of post-synaptic scaffold domain formation we proceed with studying a more detailed, truly particle-based model of scaffold aggregation by means of computer simulations. This allows us to test the influence of simplifying assumptions, like the assumed circularity of scaffold domains, and to address the possibility of multiple diffusing scaffold domains of various sizes. Most interestingly, this allows us to obtain predictions from the model on the size distribution of scaffold domains that we then compare to new data.

### Particle-based model of scaffold-protein dynamics at the membrane

In the particle-based simulations, we consider individual scaffold particles attached to the membrane that diffusive laterally at the membrane. Particles bind to each other upon encounter during their random diffusive trajectories, mimicking the homotypic interactions of scaffold proteins.

Since the details of scaffold aggregation, diffusion, and domain dynamics are yet to be described, we choose to make simple assumptions to obtain a computationally efficient model with few parameters as detailed below (see also Materials and methods for the details of our implementation).

Particles aggregate upon encounter and form clusters. We assume that particles in a cluster rearrange into a circular disc-like domain shape. (To test the influence of this assumption, we also performed a few simulations where, on the contrary, no rearrangement was allowed, as described further below.)

The disc-like clusters can themselves diffuse and aggregate. We thus need to prescribe a possible size-dependence of the diffusion constant. The Saffman-Delbrück theory [28] for thermal diffusion would predict a logarithmic size dependence of the diffusion constant arising from hydrodynamic effects (see however [29] for proteins of size comparable to the membrane thickness). This classic result may be modified by possible interactions of the scaffolding proteins with the cell cortex, non-thermal effects as well as by the more complex nature of the receptor-mediated diffusion of scaffold domains. For simplicity and to avoid introducing a characteristic size “ad hoc”, we consider a power-law size dependence

$$D\left(n\right)=D_{0}n^{-\sigma },\;\sigma \geq 0$$ (2)

where *D*(*n*) is the diffusion constant of clusters containing *n* scaffold particles and *σ* is the size-dependence exponent (*σ* = 0 when Saffman-Delbrück’s result [28] applies). In our model, *σ* is a further parameter of the system in addition to those introduced previously.

Particles are supposed to desorb into the cytoplasm with an effective rate *k*, modeled by a stochastic removal of individual particles from the membrane. We assume a constant rate *k*, irrespective of the size of the domain that particles belong to. As shown below, this assumption appears sufficient to account for present data. We also neglected lateral desorption of particles in the membrane after aggregation since binding affinity between gephyrin trimers is high. This amounts to assuming that the concentration of diffusing gephyrin particles in the membrane is large as compared to the concentration in equilibrium with the condensed scaffold phase (i.e., we neglect gephyrin “vapor pressure”). The existence of a significant lateral desorption would effectively amount to a reduced incoming flux of particles on each cluster and in an underestimate of our present fitted diffusion constant. Lateral desorption would also favor more compact aggregates [30] (see also Discussion) in the case of partial rearrangement dynamics.

The desorption of particles is balanced by an incoming flux *J* of single particles to the membrane, which ensures that the average total concentration of particles attached to the membrane *c*_0_ = *J*/*k* remains constant.

### Extrasynaptic aggregation of synaptic proteins

**Fig 2:**
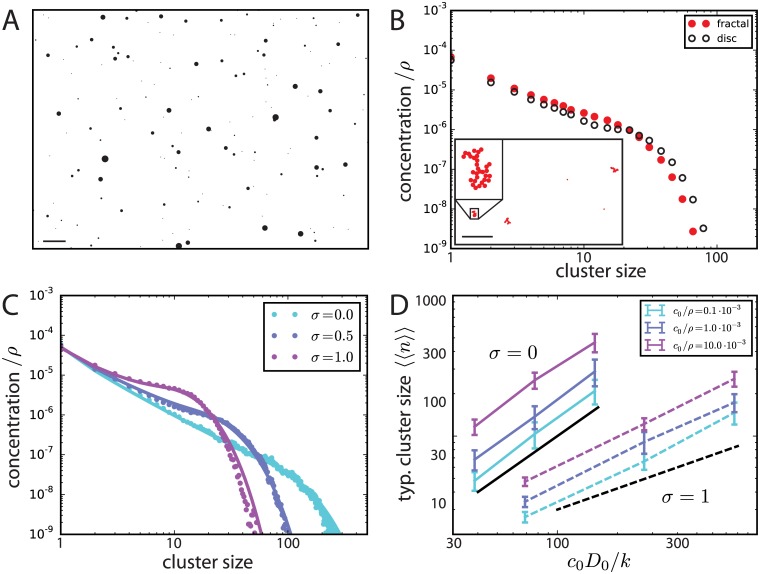
Simulations of scaffold domain formation. When not explicitly varied, simulation parameter values are *k* = 2.0 ⋅ 10^−5^*D*_0_*ρ*, *c*_0_ = 0.9 ⋅ 10^−3^*ρ*, where *ρ* = 0.77*a*^−2^ and *a* is the diameter of basic particles, and the diffusion exponent (Eq (2)) is *σ* = 0.5. A: Snapshot of scaffold domain dynamics. Scale bar 100 *a*; individual clusters are enlarged 3-fold for better visualization. B: The characteristic distribution of observed cluster sizes does not depend on the details of internal cluster structure. The cluster size distribution is shown for the simulation in panel A (black circles) with perfect particle rearrangement, i.e. disc-like clusters, and for a simulation without any particle rearrangement but otherwise identical parameters (red dots; simulation snapshot and typical cluster shown in inset). Note that the density of fractal clusters is not constant and depends on cluster size; for comparison the density *ρ* = 0.77*a*^−2^ of disc-like clusters is used here. Scale bar inset 50 *a*. C: Characteristic cluster size distributions of simulations with different diffusion exponents *σ* but otherwise identical parameters (filled circles). The data for *σ* = 0.5 corresponds to the simulation shown in A. Superposed on the simulation results are the theoretical curves obtained from the rate equations (solid lines), see text for details. D: Scaling of the typical cluster size 〈〈*n*〉〉 with *c*_0_*D*_0_/*k* for different *σ* and *c*_0_. Solid black line: 〈〈*n*〉〉 ∝ *c*_0_*D*_0_/*k*; dashed black line: 〈〈*n*〉〉 ∝ (*c*_0_*D*_0_/*k*)^1/2^.

A typical snapshot of our simulations is shown in Fig 2A. In going beyond the simplified assumption of a single scaffold non-diffusing domain, we notably find in our simulations the continuous generation of small clusters at the membrane. These clusters continue to diffuse, albeit more slowly because of their increased size, leading to an ensemble of clusters of all sizes (Fig 2A–2C). Eventually, the growth of a particular cluster is limited by the turnover of its constituent scaffold particles, analogous to the reduced model of a single domain discussed above.

As a rather general result, we find that clusters become progressively rarer with increasing size (Fig 2B and 2C). The size distribution of clusters for a diffusion constant exponent *σ* = 0.5 is shown in Fig 2B both when aggregating particles are fully rearranged into circular domains or, on the contrary, when no rearrangement is performed after aggregation. While rearrangement after clustering has a large effect on domain shape (see insert of Fig 2B), its impact on cluster size distribution is minor. This is found to be true also for other diffusion constant exponents. Since we do not focus here on scaffold domain shapes, for computational efficiency, we consider in the following only fully rearranged clusters.

The shape of the cluster size distribution is governed by the size-dependence of the cluster diffusion constant (Fig 2C). Qualitatively, when *σ* increases away from zero (i.e. the diffusion of large clusters is increasingly suppressed), the large size limit of the distribution decreases, implying a smaller range of cluster sizes. Clusters are also more evenly distributed among the available size range.

The cluster size distributions produced by particle simulations are quantitatively compared in Fig 2C to numerical solutions of Smoluchowski rate equations [25, 26, 31, 32] (see S1 Text: Rate Equations). These equations account quite accurately for the obtained distributions after fitting a single overall kinetic parameter (see S1 Text: Rate Equations and S1 Fig). For a size-independent diffusion constant, one can analytically show that the distribution is a power-law with an exponent of -3/2 and an exponential cut-off (Eq. S14 in S1 Text: Rate Equations). In the general case *σ* ≠ 0, the rate equations still allow us to numerically determine the stationary solution for the cluster size distribution (see also S1 Fig). When *σ* > 0, diffusion is progressively slower for larger and larger clusters. This reduced diffusion limits the growth of large clusters relative to smaller clusters, and the distribution is shifted towards smaller sizes with increasing *σ*. The shape progressively deviates from the power-law observed for *σ* = 0, eventually leading to the appearance of a shoulder at a smaller cut-off size beyond which clusters become again exponentially rare.

Larger clusters are rarer than smaller ones, but since they contain more scaffold particles, the majority of scaffold particles may still be found in clusters of large size. A useful quantity for characterizing such distributions is the “typical” cluster size, which corresponds to the average cluster size when clusters are weighted by the number of particles they contain. It is defined as 〈〈*n*〉〉 ≡ ∑_*n*_ *n*^2^*c*_*n*_/∑_*n*_ *nc*_*n*_, where *c*_*n*_ is the concentration of of clusters of size *n*.

The typical cluster size as a function of the biophysical parameters of diffusion and turnover is shown in Fig 2D. We find that for a given diffusion constant size-dependence exponent *σ*, the typical size essentially scales with the dimensionless parameter combination *c*_0_*D*_0_/*k* according to

$$\left\langle \left\langle n\right\rangle \right\rangle \propto {\left(\frac{c_{0}D_{0}}{k}\right)}^{\alpha },\;\alpha ≃\frac{1}{1+\sigma }\;.$$ (3)

For constant surface concentration of scaffold particles *c*_0_ and a given *σ*, the typical cluster size increases with the general diffusion constant *D*_0_ and decreases with higher effective turnover rate *k*. (Note that for constant cytoplasmic particle flux *J* onto the membrane, varying *k* also changes the total surface concentration via *c*_0_ = *J*/*k*.) In the opposite case, for varying *c*_0_ while keeping the product *c*_0_*D*_0_/*k* constant, the typical cluster size remains roughly constant; consistently, the scaling of 〈〈*n*〉〉 with *c*_0_*D*_0_/*k* does not depend on the specific value of *c*_0_ for which the simulations were done.

In principle, the scaling exponent *α* depends on the precise shape of the cluster size distribution, but a simple scaling argument rationalizes the observed relation (Eq 3). Assuming that the majority of scaffold particles are aggregated in clusters of size *N*, the concentration of these clusters is given by *c*_*N*_ ≃ *c*_0_/*N*. The average distance between the clusters thus scales as $L≃\sqrt{N/c_{0}}$. The diffusion constant of the clusters being given by *D*_*N*_ = *D*_0_*N*^−*σ*^, the timescale of their diffusive encounter scales as *T* ∝ *L*^2^/*D*_*N*_ ≃ *N*^1+*σ*^/(*c*_0_*D*_0_). For *N* to be a stable cluster size, this timescale has to match the the typical turnover time 1/*k*, otherwise clusters would either melt or grow bigger. One then immediately obtains *N* ∝ (*c*_0_*D*_0_/*k*)^1/(1+*σ*)^.

### Size distribution of gephyrin clusters

**Fig 3:**
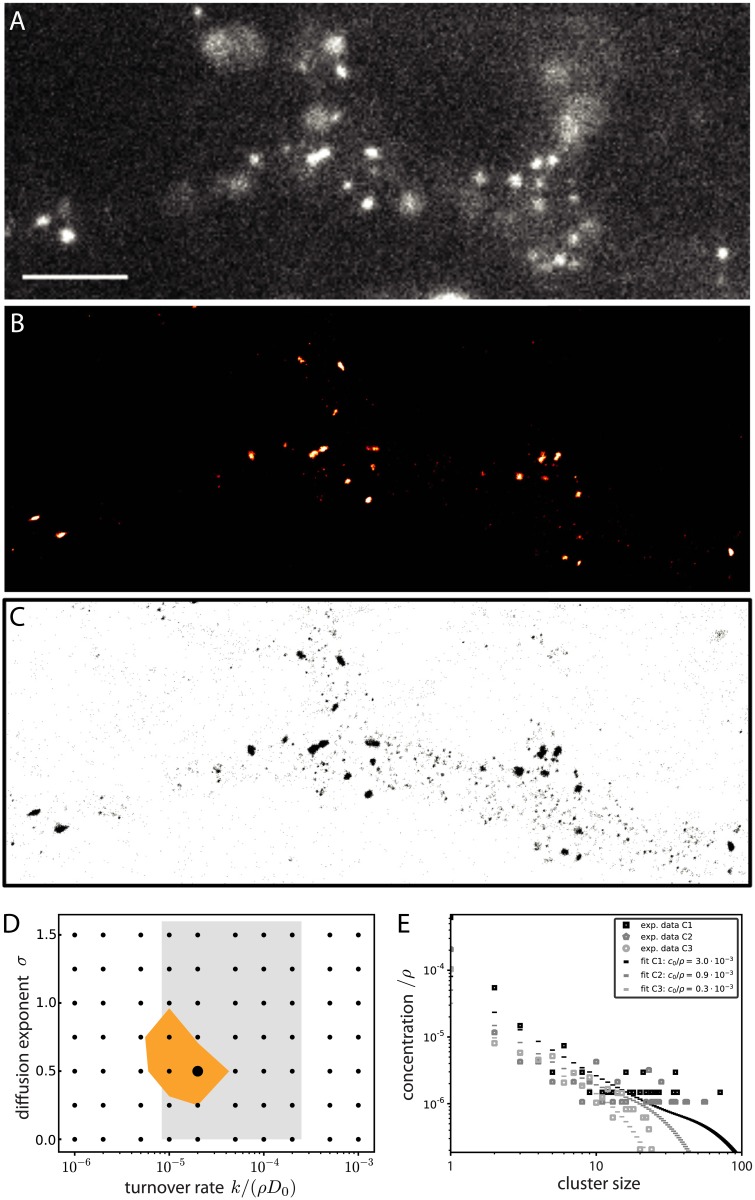
Gephyrin clusters and comparison with model distributions. A: Conventional fluorescence microscopy of spinal cord neurons expressing the mEos2-gephyrin protein. Scale bar 5 *μ*m. B: Rendered super-resolution PALM image of the same segment shown in A acquired over 20k frames at a frame rate of 20ms. C: Pointillist representation of B, where each point represents a single detection. D: Maximum-likelihood fit of the experimentally determined distributions for given parameters *k* and *σ*, with optimal *c*_0_ varying individually for each culture (Materials and methods). The highlighted parameter region (orange) corresponds to 95% probability over *N* = 10000 repeated fits of random bootstrap samples from the experimental distributions. The grey shaded region corresponds to the experimentally plausible range of *k*/*D*_0_ = 1.4 · 10^−2^ − 4.2 · 10^−1^ *μ*m^−2^. E: Experimentally determined gephyrin cluster size distributions and fitted distributions for all three cultures. Experimental concentrations were non-dimensionalized using *ρ*_trimer_ = 5000/3 *μ*m^−2^.

Our simulations predict that the sizes of scaffold domains depend on the continuous aggregation of scaffolding proteins into clusters at the membrane, which in turn supply the formation of larger domains. Because the expected distribution of cluster sizes is determined by the biophysical parameters of diffusion and turnover, these parameters can in principle be inferred from observed distributions of scaffold cluster sizes. Therefore, we experimentally determined the size distribution of gephyrin scaffolds in the neuronal membrane of cultured spinal cord neurons, both to confirm previous findings that extrasynaptic clusters exist and to provide a measurement of the relevant parameters *D*_0_/*k*, *c*_0_, and *σ*. To this aim, super-resolution microscopy on fixed neurons was used to count the number of individual gephyrin proteins in clusters (see Materials and methods, as previously published [6]). In brief, fluorescently labelled gephyrin proteins are stochastically activated at low light intensity such that the probability of concurrently activating close-by proteins is vanishingly small. The spatially separated light bursts of activated proteins can thus be localized with sub-wavelength precision. Because every labelled protein is activated one single time before being trapped in a “dark” state, the whole protein population can be imaged over the course of the experiment, and the clusters reconstructed from the recorded particle positions, see Fig 3A–3C.

The number of detections was directly associated to gephyrin cluster size [6]. We find gephyrin clusters of all sizes up to clusters of 71 trimers (see Fig 3E, S2 Fig). The largest domains are likely to be postsynaptic, 89% being found apposed to synaptic terminals in previous work [33]. Smaller gephyrin clusters should correspond to extrasynaptic clusters, consistent with the so-called nanoclusters previously visualized outside synapses with super-resolution microscopy [6].

The existence of clusters of different sizes was consistent with our predictions. Therefore, we compared the experimentally measured distributions to distributions generated by our model for different parameter combinations. Calculating the likelihood of the data for given theoretical distributions, we determined the parameters that explain best our data and which we refer to as maximum-likelihood fit of our model. Combining this analysis for all cultures (*n* = 3) by letting the total scaffold concentration, *c*_0_, vary between cultures, we obtain a global estimate for the diffusion exponent, *σ* = 0.5, and the ratio of the diffusion constant over the particle turnover rate, *D*_0_/*k* = 30 *μ*m^2^, see Fig 3D. The total scaffold concentration varies among cultures from *c*_0_ = 0.5 − 5 *μ*m^−2^, see Fig 3E.

### Cluster dynamics and size fluctuations of scaffold domains

**Fig 4:**
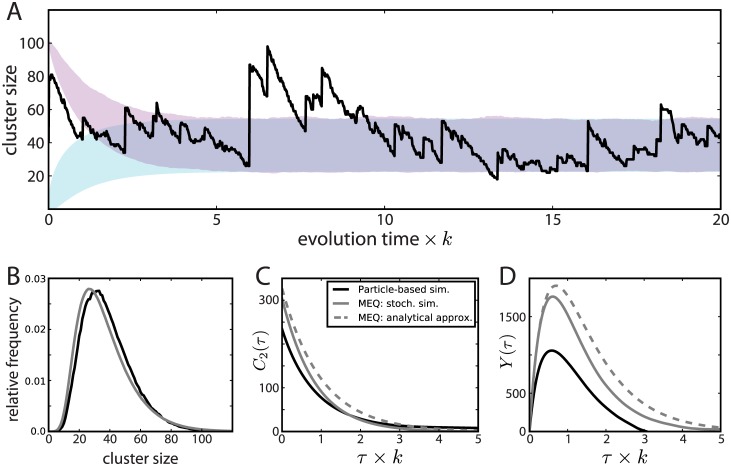
Predicted size evolution of individual clusters. A: Average cluster size (mean ± SD) evolution for clusters of an initial size of 2 (light blue) and 100 (light red) particles, respectively, averaged over independent simulated cluster size trajectories (*n*_2_ = 50 ⋅ 10^6^, *n*_100_ = 4000) sampled from simulations. A typical cluster size trajectory is also shown (dark solid line). Simulation parameters are identical to Fig 2A. B: The distribution of cluster sizes at long times. Large clusters persist for a long time and explore the size distribution shown here due to particle addition by fusion with other clusters and particle removal by desorption. The distribution obtained from simulated trajectories (*n* = 1000) using the master equation (MEQ) approach (grey solid line, see text for details) is slightly different as spatial correlations are ignored. C: Autocorrelation, *C*_2_(*τ*) = 〈*n*(*t* + *τ*)*n*(*t*)〉 − 〈*n*〉^2^, of the cluster size *n*(*t*) obtained from full particle simulations (black solid line) and from the MEQ approach (solid grey line). The analytical prediction of an exponential decay with characteristic rate *k* is also shown (dashed grey line). D: The function *Y*(*τ*) = 〈*n*(*t* + *τ*)*n*(*t*)^2^〉 − 〈*n*(*t* + *τ*)^2^*n*(*t*)〉 is shown (solid black line). The fact that *Y*(*τ*) is non-zero implies that the dynamics is not invariant under time reversal and therefore out-of-thermodynamic-equilibrium. The approximation *Y*(*τ*) ∝ exp(−*kτ*)[1 − exp(−*kτ*)] is also shown (dashed grey line) as well as the function *Y*(*τ*) generated by the MEQ approach (solid grey line) (see text and S1 Text: Rate Equations for details).

Our simulations allow us to address the temporal size fluctuations of scaffold clusters and their stability over time. The size trajectory of an individual cluster is shown in Fig 4A. It fluctuates around a well-defined mean size, alternating between stochastic increases due to fusion with other clusters, and relatively smooth decreases on a characteristic timescale *τ*_fluc_ due to the continuous particle loss into the cytoplasm. This behavior is typical of all followed clusters. In particular, clusters of small initial sizes grow by fusion with other clusters and fluctuate around the mean size after a few *τ*_fluc_ (Fig 4A, light blue lines). This leads the distribution of sizes explored by a cluster over time, shown in Fig 4B, to differ from the instantaneous distribution of all clusters at the membrane at a given time (Fig 3E), with a suppression of small cluster sizes. The cluster size autocorrelation *C*_2_(*τ*) = 〈*n*(*t* + *τ*)*n*(*t*)〉 − 〈*n*〉^2^ allows a quantification of the fluctuation dynamics around the mean cluster size. It is shown in Fig 4C both for the particle-based simulations and for simulated trajectories based on a master equation approach (see S1 Text: Rate Equations). A simplified description of the latter approach provides an explicit expression for *C*_2_(*τ*) and predicts that *τ*_fluc_ is set by the scaffolding protein turnover rate, *τ*_fluc_ ≃ 1/*k* (see S1 Text: Rate Equations) in good quantitative agreement with the simulations.

The cluster size variation over time depicted in Fig 4A is clearly not invariant under time reversal. It is interesting to note that it is a direct signature of the out-of-thermodynamic-equilibrium nature of the process considered since fluctuations at thermodynamic equilibrium should not allow to determine the arrow of time. One quantitative measure of the out-of-equilibrium nature of the underlying dynamics is provided by the third moment *Y*(*τ*) = 〈*n*(*t* + *τ*)*n*(*t*)^2^〉 − 〈*n*(*t* + *τ*)^2^*n*(*t*)〉 [34, 35] which by construction vanishes for systems that are invariant under time reversal. The non-trivial function *Y*(*τ*) obtained from cluster size time trajectories is shown in Fig 4D, together with approximations based on the master equation approach (see S1 Text: Rate Equations).

## Discussion

We propose a model of scaffold domain formation and maintenance. It is based on

1. the delivery of scaffolding proteins at the plasma membrane,
2. receptor-mediated scaffold diffusion at the plasma membrane, and
3. scaffold growth upon encounter and multimerization with other scaffolds.

The counterbalance of this cluster growth process by the continuous removal of scaffolding proteins leads to a stabilization of a maximal domain size as well as a to a stationary distribution of scaffold clusters of different sizes. In a previous work [6], a population of small extrasynaptic clusters had been shown to coexist with large synaptic clusters, but the sizes of the former had not been precisely quantified. We have here provided new super-resolution microscopy data which shows that post-synaptic clusters assume a continuous range of sizes from gephyrin trimers up to sizes characteristic of synaptic domains. This agrees with a characteristic signature of the aggregation-removal dynamics proposed to underlie their formation. Comparison of experimental data with model size distributions allowed us to refine the biophysical parameters of this dynamics. Moreover, we found that the existence of diffusing extrasynaptic clusters has important consequences for the size fluctuations of synaptic domains, as the former may fuse with the latter. Our simulations have been focused on the dynamics of these extrasynaptic clusters. When one of them is followed over time, it reaches on the timescale of an hour the large size end of the domain size distribution and fluctuates around it, as shown in Fig 4A. In the simplest picture, the synaptic domains are simply the product of this formation process and their size distribution is as given by Fig 4B. However, this is most probably an oversimplification since the properties of synaptic scaffold domains certainly differ from extrasynaptic ones. For instance, we expect them to diffuse even less than large extrasynaptic scaffold domains. This would reduce their probability of encounters with other domains and consequently their size (simulations with one fixed domain show a mean size reduction of 33% as compared to Fig 4B).

In recent years, thermodynamic phase transitions and phase separation have been found to underlie the formation of various cellular membrane-less structures [36], most recently the PSDs of excitatory synapses [37]. One feature of thermodynamic phase separation is that the growth of the condensed phase is only limited by the depletion of the condensing component. The size of the formed structure is thus determined by the size of its “container” as well as the total number of molecular components enclosed in the latter [38]. In cells, the container can be the whole cell or one of its sub-compartments, such as a spine for an excitatory PSD. Non-equilibrium phase separation may allow cells to avoid this constraint and to form localized structures of definite size, such as PSDs, irrespective of the size of the compartment in which they form, namely the whole neuron membrane for the majority of inhibitory synapses. Aggregation with removal or turnover has been proposed in various cellular contexts [21, 23] besides the one considered here. It is tempting to suggest that it is a specific, easily implementable, out-of-thermodynamic-equilibrium phase separation mechanism generally used by cells to form localized structures.

Our results expand previous works considering the non-equilibrium dynamics of receptors and scaffolds at the plasma membrane. While Haselwandter et al. [18, 19] considered general continuous equations for the interaction of receptors and scaffold proteins, they analyzed a different dynamical regime. Their mathematical analysis showed that domains of a characteristic size can be obtained via a Turing-like instability. In this case, the cooperative binding of scaffold proteins at synaptic domains is limited by receptors diffusing away from a domain and steric repulsion between receptors. However, it is unclear whether these assumptions are all verified in neurons, as for example, the receptor density appears to be far below saturation on synaptic domains [39, 40]. Moreover, in this Turing regime, a continuous range of domain sizes would not be expected. Burlakov et al. [20] proposed a model based on receptor aggregation and removal similar in spirit to the one described in Fig 1, but they did not consider scaffolding proteins nor cluster size distributions.

In the present study, we have studied a simple model of PSD formation, with a minimal number of parameters, most of which are determined by available data. Nonetheless, several of our assumptions may need to be refined when further data become available. In our model, receptors act mainly as carriers that enable the lateral diffusion of scaffold particles and domains along the cell membrane. Our single rate *k* for scaffold removal from the membrane accounts both for scaffold dissociation from receptors and for the endocytosis of receptor-scaffold complexes. This appears sufficient at present but receptors may need to be accounted for independently of scaffolds if one wishes to describe these two processes more precisely. A description with multiple molecular species would also be needed to describe the concentration of receptors on scaffold domains, its dependence on scaffold-receptor affinity as well as an influence of receptors of scaffold domain stability. A dependance of scaffold removal on scaffold domain size may also need to be included.

We have mainly considered the limit where scaffold domains rearranged themselves very efficiently and adopt a spherical compact shape. We have shown that this does not have significant consequences for cluster dynamics and size distributions by considering the other extreme limit of negligible cluster reshaping after scaffold aggregation, see Fig 2B. However, the shape of the individual scaffold clusters does depend on the rearrangement of scaffold particles within a domain or absence thereof (see e.g. inset Fig 2B). It is well-known that in the absence of recycling, clusters which grow by diffusion are prone to shape instabilities and that shapes are sensitive to changes in the dynamics. A prototypical example is provided by the classical diffusion-limited aggregation (DLA) model [41], where clusters grow by irreversible aggregation of very diluted single monomers without rearrangement. Such clusters assume isotropic ramified shapes with a fractal dimension *d*_f_ ≃ 1.7 for monomer diffusion in two dimensions (*d* = 2) as considered here. Preferred directions of attachment or crystalline anisotropy make the clusters anisotropic as commonly observed with snowflakes [42]. When not only monomers but also clusters diffuse and aggregate—a case known as cluster-cluster aggregation—the produced cluster shapes are different with a fractal dimension of *d*_f_ ≃ 1.45 [30]. Growth in a monomer solution of finite concentration *c*_0_ renders DLA clusters compact above a characteristic length that depends on *c*_0_ [30]. Removal of monomers furthermore introduces a “diffusion length” $\lambda =\sqrt{D_{0}/k}$ above which clusters also cease to be fractal. With the parameters of Fig 3D, λ ≃ 5 *μ*m is much larger than the linear size of synaptic domains. It will therefore be an interesting task for future studies, both experimental and theoretical, to investigate scaffolding protein removal from scaffold domain and the modes of scaffold domain rearrangements and link them to the observed shapes which appear neither circular nor fully fractal.

The strength of a synapse is one of its key properties since it controls the efficacy of information transmission between neurons. However, when synapses are monitored over the course of days, they undergo large fluctuations in size and molecular content. This holds for neurons cultured *in vitro*, in absence of learning protocols, and even in absence of activity [43]. A phenomenological model, based on a mixed additive-multiplicative stochastic process [44], has been found to account well for the recorded size fluctuations of excitatory [45] as well as inhibitory [46] synapses. Interestingly, we found here that the existence of diffusing extrasynaptic clusters may provide a mechanistic explanation for similar fluctuations of scaffold domain sizes over shorter periods. The predicted distribution of fluctuating domain sizes is skewed (see Fig 4B) and qualitatively similar to those reported (see e.g. Fig 4 in ref. [46]).

Besides spontaneous fluctuations, the elucidation of the mechanisms by which synaptic strength is specifically modified remains a key challenge. The role of lateral diffusion of receptors in the plasma membrane in mediating receptor number changes at the synapse has started to be considered in the context of synaptic plasticity [1, 8] and synaptic strength homeostasis [47]. Both the lateral mobility of GlyR in the cell membrane and its binding affinity for gephyrin [48] can be regulated. In the framework of the model that we propose, this would induce changes not only in the receptor concentration on scaffold domains, but also in the size of the scaffold domain themselves, by modifying the lateral flux of scaffolding proteins onto a domain. The removal time of scaffold proteins is in our model another important parameter that could be acted upon to regulate scaffold domain size. In this context, it can be noted that smaller gephyrin domain sizes were reported in one-week old organotypic cultures as compared to four-weeks old cultures [10] while, correlatively, the mean residence time of gephyrin in scaffold domain was measured to be shorter in one-week old cultures than in four-weeks old cultures [10]. We are confident that a biophysically-rooted model such as the one we propose here offers new perspectives on synaptic dynamics, homeostasis and plasticity that it will be useful to explore in future works.

## Materials and methods

### Simulations

As described in the Results, we implemented two versions of the basic model, which correspond to two limits of particle rearrangement within clusters. In model A, particles within a given cluster immediately rearrange into a disc with a radius given by the number of particles *n* and a typical particle number density in clusters *ρ*, $R=\sqrt{n/(\pi \rho )}$. In model B, individual particles do not rearrange, and clusters form fractal-like structures. Both models essentially produce the same distribution of cluster sizes, see Fig 2B. Except explicitly stated otherwise, all simulation results presented were obtained with model A for reasons of reduced computational complexity.

In the simulations, all relevant quantities and parameters are expressed in the units of length *l*_0_ = *a* and of time *t*_0_ = *a*^2^/*D*_0_, respectively, where *a* is the diameter and *D*_0_ the diffusion constant of an individual particle. Initially, *N* = *c*_0_*L*^2^ particles are randomly distributed in space in a square of side length *L*, and we use periodic boundary conditions throughout. Particle diffusion and turnover are approximated in discrete time steps Δ*t* = 0.02 small compared to the characteristic time *t*_0_ of particle diffusion. During each step, we first update particle and cluster positions, indexed by *i* = 1, …, *M*, by random increments according to the respective diffusion constant *D*_*i*_, i.e., x and y increments drawn from a normal distribution with variance 2Δ*tD*_*i*_ and vanishing mean. Particles or clusters that overlap afterwards fuse to give a cluster of size *n*_*i*_ + *n*_*j*_, where *n*_*i*_ and *n*_*j*_ are the sizes of the fusing clusters/particles, and an accordingly updated radius. Eventually, we draw the number of desorbed particles per cluster within one time step from a binomial distribution with *n*_*i*_ independent draws each having probability Δ*t* *k* and reduce the cluster sizes and radii accordingly. For simplicity, we keep the total number of particles in the simulation constant and insert as many new, randomly distributed particles as were removed.

Simulations were generally carried out with *N* ≃ 10^4^ particles, with the box size *L* varying correspondingly for different concentrations *c*_0_. In our simulations, we chose a cluster density of *ρ* = 0.77*a*^−2^ that corresponds to the packing fraction of a hexagonal arrangement; however, the comparison with simulations of fractal clusters shows that the results do not depend much on this choice.

### Reagents

Unless otherwise noted, all reagents were purchased from Sigma-Aldrich (St. Louis, MO) or Life Technologies/Molecular Probes (Carlsbad, CA).

### Lentivirus

A lentivirus encoding mEos2-Gephyrin was produced by cotransfecting the lentiviral backbone plasmid (FUGW) encoding the mEos2-Gephyrin construct (5 *μ*g) along with the pMD2.G envelope (5 *μ*g) and pCMVR8.74 packaging (7.5 *μ*g) plasmids (Addgene, Cambridge, MA) into HEK293T cells using lipofectamine 2000 (60 *μ*l). Transfection was performed in 10 cm plates once the cells reached 80% confluence. Supernatant containing lentivirus was collected 48 h after transfection, filtered through a 0.45 *μ*m-pore-size filter, aliquoted, and stored at −80°C.

### Cell culture and infection

All experiments were performed on dissociated spinal cord neuron cultures prepared from Sprague-Dawley rats (at E14). Experiments were carried out in accordance with the European Communities Council Directive 2010/63EU of 22 September 2010 on the protection of animals used for scientific purpose and our protocols were approved by the Charles Darwin committee in Animal experiment (Ce5/2012/018). Neurons were plated at a density of 6.3 × 10^4^ cells/cm^2^ on 18 mm coverslips pre-coated with 70 *μ*g/ml poly-D,L-ornithine and 5% fetal calf serum. Cultures were maintained in neurobasal medium containing B-27, 2 mM glutamine, 5 U/ml penicillin, and 5 *μ*g/ml streptomycin at 37°C and 5% CO_2_. Neurons were infected at 7 days in vitro (DIV) with a recombinant lentiviral vector expressing the mEos2-Gephyrin construct.

### PALM imaging

Photoactivated localization microscopy (PALM) was performed at DIV 14 − 17 on neurons fixed with 4% paraformaldehyde and 1% sucrose (10 min). All imaging experiments were performed on an inverted Nikon Eclipse Ti microscope with a 100× oil-immersion objective (N.A. 1.49), an additional 1.5× lens, and an Andor iXon EMCCD camera. Super-resolution movies were acquired at 20 ms frame rate under continuous illumination with activation (405 nm) and excitation (561 nm) lasers for a total of 20000 frames (6.7 minutes). Activation density was kept steady by manually increasing the activation laser intensity over time. Conventional fluorescence imaging was performed with a mercury lamp and filter sets for the detection of preconverted mEos2 (excitation 485/20, emission 525/30). The z-position was maintained during acquisition by a Nikon perfect focus system.

### Single molecule detection and PALM reconstruction

The x and y coordinates of single molecule detections from each image frame were determined using an adapted version of the multiple-target tracking algorithm [49] as described previously [50]. The point spread function (PSF) signals emitted by single fluorophores were fit with a 2D Gaussian distribution. Drift in the x/y plane was corrected by calculating the relative movement of the center of mass of multiple synaptic gephyrin clusters (more than 4 per field of view), throughout the acquisition, using a sliding window of 3000 − 6000 frames. Activations were clustered with using a single-link clustering, with a minimal distance of 50nm. Single temporal bursts in low density regions were measured for location accuracy and double counting, allowing for the counting of molecules within clusters. Regions of interests were selected by hand from fluorescent images such that only clusters within dendrites were selected for size concentration analysis. Analysis was performed on (*n* = 3) cultures.

### Fits of simulated cluster size distribution to experimental data

To compare the experimentally determined cluster size distributions to the predicted distributions for a given set of parameters *S*, we determined the likelihood of the data according to

$$L\left(\left\{{\#}_{i}\right\};S\right)=\prod_{i=2}^{i_{\text{max}}}\frac{{n_{i}}^{{\#}_{i}}e^{-n_{i}}}{{\#}_{i}!}\;,$$ (4)

where *n_i_* = *A*_exp_*c_i_*(*S*) is the predicted count of clusters of size *i* in the observed surface area *A*_exp_ for given parameters *S*, and #_*i*_ is the actually determined count. The likelihood is computed including sizes up to the largest observed cluster size *i*_max_. For each culture (*n* = 3), we determined the likelihood over a grid of parameter values, varying turnover rate, diffusion size-dependence exponent, and total concentration. Assuming that the latter may vary between cultures, we determined the joint likelihood $L_{\text{tot}}=\prod_{j}\;L_{j}$ over all three cultures *j* = 1, 2, 3 for parameters (*k*, *σ*), choosing the most likely *c*_0_ for each (*k*, *σ*) independently for each culture. Maximum-likelihood fits of the individual clusters are shown in S3 Fig. In order to compare the experimental and simulation results, we adimensionnalized the concentrations by the density of scaffold particles in the clusters, (*ρ* = 5000/3 *μ*m^−2^) for experiments and (*ρ* = 0.77*a*^−2^) for simulations, respectively. We assumed that gephyrin exists predominantly in trimer form and thus considered gephyrin trimers as the single-particle element in the cluster size count. We furthermore restricted the comparison of predicted and actual distributions to cluster sizes of *i* ≥ 2, as counts of smaller clusters are more affected by impurities and measurement noise. The predicted particle concentrations were determined from the stationary solutions to the rate equations, see S1 Text: Rate Equations. Confidence regions corresponding to 95% probability of the optimal fit parameters were obtained by a bootstrapping technique using repeated resampling (*N* = 10000) of the observed clusters for each of the three cultures, and applying the above fit procedure to the resampled cluster size distributions.

## Supporting information

S1 Text Supporting analytical calculations. The text file includes the sections “Single scaffold domain” and “Rate equations”.
(PDF)

S1 Fig Rate-equation description of scaffold-cluster aggregation. Comparison and match of the cluster size distributions obtained from the rate-equation description to the full simulations.
(EPS)

S2 Fig Fluorophore detections per cluster. Raw data of the fluorophore detections from which the gephyrin cluster sizes were obtained.
(EPS)

S3 Fig Maximum-likelihood fits for individual cultures. Fits of data and confidence regions using bootstrap resampling (*N* = 10000) performed independently for each culture.
(EPS)
