# Supplementary material for: An aggregation-removal model for the formation and size determination of post-synaptic scaffold domains
Source: PLoS Comput Biol. 2017 Apr 24;13(4):e1005516. doi: 10.1371/journal.pcbi.1005516 (PMC5421815; doi:10.1371/journal.pcbi.1005516)
Supplement: S1 Text — The text file includes the sections “Single scaffold domain” and “Rate equations”. (PDF) [file pcbi.1005516.s001.pdf]

SI – An aggregation-removal model for the formation and size determination of post-synaptic scaffold domains

Jonas Ranft,[1,*] Leandro G. Almeida,[2,*] Pamela L. Rodriguez,[2] Antoine Triller,[2] and Vincent Hakim[1]

[1]*Laboratoire de Physique Statistique, CNRS, Ecole Normale Supérieure, PSL Research University, Université Pierre et Marie Curie, Université Paris Diderot, Paris, France*

[2]*IBENS, Ecole Normale Supérieure, PSL Research University, CNRS, INSERM, Paris, France*

[*] equal contribution

I. SINGLE SCAFFOLD DOMAIN

We consider the simplest case of a single scaffold domain surrounded by units, i.e. bound receptor-scaffold complexes, diffusing in the neuronal membrane. We suppose that delivery of scaffold proteins at the cell membrane is not spatially specific and simply creates new diffusing units with a rate J per unit membrane area. These diffusing units disappear when scaffolds detach from diffusing receptors at a rate k. The concentration c of the diffusing units is thus governed by the equation

$$\partial_t c = D_0 \nabla^2 c - k\,c + J\,, \tag{S1}$$

Far from the scaffold domain, the concentration c reaches its asymptotic value $c_\infty = J/k$. At the boundary of the scaffold domain, when the kinetics of unit attachment and detachment is fast compared to the diffusion timescale $t_D = (c_\infty D_0)^{-1}$, c is given by the equilibrium concentration $\bar{c}_{\text{eq}}$. The concentration $\bar{c}_{\text{eq}}$ is determined by the binding free energies of scaffold complexes and we assume that is is small enough to be negligible, $\bar{c}_{\text{eq}} \simeq 0$. For a stationary circular synaptic domain of radius R, the steady concentration is circularly symmetric and reads

$$c(r) = c_\infty \left[1 - \frac{K_0(r/\lambda)}{K_0(R/\lambda)}\right], \tag{S2}$$

where λ is the diffusion length, $\lambda = \sqrt{D_0/k}$, and $K_0(x)$ is the modified Bessel function of order 0 which tends to 0 as x tends to infinity [1]. The associated impinging flux ϕ_{on} of diffusing units onto the domain is given by

$$\phi_{\text{on}} = 2\pi R\, D_0\, (\partial_r c)|_R = 2\pi D_0\, c_\infty \frac{R}{\lambda}\frac{K_1(R/\lambda)}{K_0(R/\lambda)} \tag{S3}$$

An outgoing flux ϕ_{off} of scaffolding proteins from the scaffold domain is produced from the recycling of scaffold proteins into the cytoplasm at a rate $k_{s,\text{off}}$,

$$\phi_{\text{off}} = \pi R^2 \rho k_{s,\text{off}}, \tag{S4}$$

where ρ is the concentration of scaffolding proteins in a scaffold domain. For a stationary domain, the incoming and outgoing fluxes should be equal. The balance of ϕ_{on} and ϕ_{off} determines the size R by the implicit equation

$$\frac{k}{k_{s,\text{off}}}\frac{c_\infty}{\rho} = F(R/\lambda), \text{ with } \quad F(x) = xK_0(x)/[2K_1(x)]. \tag{S5}$$

The function F vanishes at x=0 and grows monotonically with $F(x) \sim x$ for large x. Inversion of Eq. (S5) provides

$$N = \pi R^2 \rho = \frac{\rho D_0}{k} \Phi\left(\frac{k}{k_{s,\mathrm{off}}} \frac{c_\infty}{\rho}\right), \tag{S6}$$

with

$$\Phi(x) = \pi [F^{-1}(x)]^2 \,, \tag{S7}$$

which is plotted in Fig. 1C,D with $k_{s,\mathrm{off}} = k \equiv k$.

II. RATE EQUATIONS

A. General framework

We can describe the aggregation of scaffold clusters in an approximate manner by a set of rate equations that describe the deterministic evolution of the concentrations $c_n(t)$ of clusters with sizes n due to fusion and turnover. Without turnover, these equations are also known as the Smoluchowski coagulation equations, initially put forward to describe the aggregation of small gold particles in aqueous solution [2]. In our case, they read

$$\frac{\mathrm{d}c_1}{\mathrm{d}t} = J + k\,(2c_2 - c_1) - \sum_{k=1}^{\infty} K_{1,k} c_1 c_k \,, \tag{S8a}$$

$$\frac{\mathrm{d}c_n}{\mathrm{d}t} = k\,[(n+1)c_{n+1} - n c_n] - \sum_{k=1}^{\infty} K_{n,k} c_n c_k + \frac{1}{2} \sum_{m=1}^{n-1} K_{m,n-m} c_m c_{n-m} \tag{S8b}$$

$n = 2, 3, \ldots$, where $K_{n,m} = K(D_n + D_m)$ is an effective fusion kernel that accounts for diffusive encounters between clusters of size n and m with diffusion constants D_n and D_m, respectively. Note that we neglect any additional dependence of the fusion kernel on cluster size for simplicity; our simulations show that this is indeed a reasonable assumption. However, the scalar factor K varies with the total scaffold surface concentration c_0, see Fig. S1A. When using the correspondingly fitted value of K, the stationary size distributions predicted by Eqs. (S8) match the size distributions obtained by particle-based simulations, see Fig. S1B,C.

In general, the set of equations (S8) can also be supplemented with terms that account for the fragmentation of clusters, a process that we neglect here. Equations of this and similar

type have been studied in a variety of contexts [3, 4]. In the context of lipid raft formation in membranes and assuming size-independent diffusion $D_n = D_0$, different recycling regimes have been investigated using this approach [5].

In order to compare the predicted dynamics to our simulations, we impose that the total number density of particles at the membrane remains constant, $\sum_n nc_n(t) = c_0$, which amounts to a condition on the flux of monomers onto the membrane, $J = kc_0$. Equivalently, for a given J the total number density of particles at the membrane in the stationary state is given by $\sum_n nc_n^* = J/k$. Starting with the initial condition $c_n(0) = c_0\delta_{n1}$, i.e., from purely monomeric scaffold particles, we obtain the time evolution of the cluster size distribution by numerically integrating Eqs. (S8). The system eventually reaches a stationary state with a characteristic distribution c_n^* that depends on the turnover rate and the diffusivity D_n of clusters, as observed in our simulations. When using the correspondingly fitted value of K, the stationary size distributions predicted by Eqs. (S8) match precisely the size distributions obtained by particle-based simulations, see Fig. S1C. In line with that observation, the predicted scaling of the typical cluster size $\langle\langle n\rangle\rangle$ with turnover rate k and σ, where $D_n = D_0 n^{-\sigma}$, matches precisely the observed scaling in the particle-based simulations, see Fig. S1B. We therefore use the stationary distributions computed from Eqs. (S8) for the fits of the experimental distributions. This avoids to run long simulations for every parameter combination against which we compare our experimental data, see also Materials and Methods in the main manuscript.

B. Analytical steady-state solution for size-independent cluster diffusion

In the limit of size-independent cluster diffusion, $\sigma = 0$, one can furthermore solve Eqs. (S8) analytically for the characteristic distribution c_n^* in the stationary state. The equations now read

$$0 = J + k\,(2c_2 - c_1) - 2D \sum_{k=1}^{\infty} c_1 c_k\,, \tag{S9a}$$

$$0 = k\,[(n+1)c_{n+1} - nc_n] - 2D \sum_{k=1}^{\infty} c_n c_k + D \sum_{m=1}^{n-1} c_m c_{n-m} \qquad (n = 2, 3, \ldots)\,, \tag{S9b}$$

where we dropped the asterisks for better readability and defined $D = KD_0$. By introducing the generating function

$$G(s) = \sum_n s^n c_n$$

and summing over equations (S9) multiplied by s^n, we obtain an equation for $G(s)$,

$$0 = J(s-1) + D[G(s) - G(1)]^2 - k(s-1)G'(s)\,. \tag{S10}$$

The above equation allows us already to determine the typical cluster size: Taking the third derivative of (S10) at $s = 1$, and using $G''(1) = \langle\langle n\rangle\rangle c_0 - c_0$, one immediately obtains

$$\langle\langle n\rangle\rangle = 1 + \frac{c_0 D}{k}\,. \tag{S11}$$

To actually solve for the stationary distribution, we non-dimensionalize $G(s)$ using the transformation $g(s) = \sqrt{D/J}\,[G(s) - G(1)]$, which leads to the following equation for $g(s)$:

$$\epsilon g' = 1 - \frac{g^2}{1-s}\,,$$

where $\epsilon = \sqrt{\frac{k}{c_0 D}}$. In the limit of vanishing ϵ, i.e., $\langle\langle n\rangle\rangle \gg 1$, one finds that g behaves as $g(s) \simeq \sqrt{1-s}$, which allows us to infer the scaling of c_n with n. The relation $c_n = \sqrt{J/D}\,g^{(n)}(0)/n!$ implies

$$c_n \sim n^{-\frac{3}{2}} \tag{S12}$$

up to n for which the approximation of g by the series expansion of $\sqrt{1-s}$ breaks down.

Singularity analysis further tells us that if the radius of convergence ρ of a generating function is larger than 1, c_n is eventually dominated by an exponential decay ρ^{-n} for large n, with the subdominant scaling being given by the type of the singularity at ρ [6]. Introducing the function $f(x) = 2g(s)/\epsilon$ with $s = 1 + (\epsilon x/2)^2$, we obtain an equation for f that we can explicitly solve:

$$0 = xf' - x^2 - f^2\,,$$

the solution of which is given by

$$f(x) = \frac{xJ_1(x)}{J_0(x)}\,. \tag{S13}$$

J_1 and J_0 denote the Bessel functions of first and zeroth order, respectively. In principle, one can now compute all c_n from the solution for $G(s)$, although this becomes computationally hard for increasing n. Here, we content ourselves with the determination of the scaling of

c_n for large n. The radius of convergence of the generating function $G(s)$ corresponds to the singularity of $f(x)$ at the first zero x_0 of $J_0(x)$ with $x_0 \simeq 2.4048$. This gives $\rho = 1+(\epsilon x_0/2)^2$. In the limit of $\epsilon \ll 1$, or large $\langle\langle n \rangle\rangle$, the concentration c_n thus decays for large n as

$$c_n \sim n^{-\frac{3}{2}} \exp\left(-n/n_{\mathrm{dec}}\right) , \tag{S14}$$

with $n_{\mathrm{dec}} = (2/x_0)^2 \langle\langle n \rangle\rangle = 0.692 \ldots \langle\langle n \rangle\rangle$.

One can derive analytic expressions also in the limit of $\sigma \to \infty$, i.e., in the case when only monomeric particles diffuse. In this case, $\langle\langle n \rangle\rangle$ grows only sub-logarithmically with Kc_0D_0/k, and macroscopic cluster sizes are not to be expected in this scenario, consistent with our simulations.

C. Stochastic evolution of single domains

In order to investigate the size evolution of individual domains in the stationary state, one can interpret the terms on the right-hand side of Equations (S8) as transition probabilities $P_{n\to m}$ from size n to size m during a small time $\mathrm{d}t$. In this master equation approach, the respective size transition probabilities are given by,

$$P_{n\to m} = \begin{cases} K_{n,m-n} c_{m-n}\,\mathrm{d}t & \text{for } m > n \\ kn\,\mathrm{d}t & \text{for } m = n-1 \\ 0 & \text{for } m < n-1 \\ 1 - \sum_{k\neq n} P_{n\to k} & \text{for } m = n. \end{cases} \tag{S15}$$

The size trajectory $n(t)$ of a cluster with an initial size n_0 at $t = 0$ can be simulated using the standard Gillespie algorithm for stochastic dynamics [7]. The results shown in Fig. 4B,C in the main manuscript are obtained by averaging over a large ensemble of such simulated trajectories in the corresponding stationary state. Note that a similar approach has been taken by Vagne et al. [8] to study the effect of lateral confinement on the clustering dynamics of continuously recycled membrane components.

A simplified description of the stochastic dynamics of an individual domain allows one to obtain explicit formulas for the domain size autocorrelation in time and the function $Y(t)$ shown in Fig. 4C,D. One can write the evolution of the size $n(t)$ of one domain as a function

of time t as

$$\frac{d}{dt}n = -k\,n + \sum_m \sum_i m\,\delta[t - t_i(m)]\,. \tag{S16}$$

In the first term on the right-hand-side (r.h.s.) of Eq. (S16), the stochastic particle loss with rate k has been approximated by the corresponding deterministic size decay. This should be accurate as soon as the cluster is not small ($n \gg 1$) which is here the regime of interest. The second term on the r.h.s. describes the sudden size increase of the cluster when it encounters a cluster of size m at times $\{t_i(m)\}$. We consider the regime where the distribution of diffusing clusters is stationary. We suppose that the times $\{t_i(m)\}$ are given by homogeneous Poisson rates $r(m)$, i.e., the rate of diffusive encounters with a cluster of size m is independent of time and only depends on the cluster size m (see below).

The simplified description (S16) allows one to easily calculate the time-averaged moments of the domain size $n(t)$. First, $n(t)$ can be explicitly written as

$$n(t) = \sum_m \sum_{t_i(m)<t} m\,\exp[-k(t - t_i(m))]\,. \tag{S17}$$

Then, the average domain size $\langle n(t)\rangle$ is obtained by averaging over the stochastic times $t_i(m)$,

$$\langle n(t)\rangle = \sum_m \int_{-\infty}^{t} dt' r(m)\,m \exp[-k(t - t')] = \frac{1}{k}\sum_m r(m)\,m\,. \tag{S18}$$

The average size $\langle n(t)\rangle$ is independent of time and we simply denote it by $\langle n\rangle$ in the following. The temporal autocorrelation of the domain size is obtained in a similar way from (S17),

$$\langle n(t)n(t')\rangle = \langle \sum_m \sum_{m'} \sum_{t_i(m)<t} \sum_{t_j(m')<t'} m\,m'\,\exp[-k(t - t_i(m))]\exp[-k(t' - t_j(m'))]\rangle\,. \tag{S19}$$

On the r.h.s. of Eq. (S19), terms where the fusion times $t_i(m)$ and $t_j(m')$ are different, average to $\langle n\rangle^2$ whereas the remaining ones give a true second-order contribution,

$$\begin{aligned}\langle n(t)n(t')\rangle - \langle n\rangle^2 &= \sum_m \int_{-\infty}^{\min(t,t')} dt''\,r(m)\,m^2 \exp[-k(t+t') + 2kt''] \\ &= \left[\frac{1}{2k}\sum_m r(m)\,m^2\right] \exp(-k|t - t'|)\,. \end{aligned} \tag{S20}$$

The autocorrelation function is symmetric in the two times t and t' and thus invariant under time reversal. One needs to compute a third-order time-asymmetric function to witness

the non-invariance of the process (S16) under time reversal and its out-of-thermodynamic equilibrium nature [9]. One possible choice [10] is provided by

$$Y(t-t') = \langle n(t)n^2(t')\rangle - \langle n^2(t)n(t')\rangle\,. \tag{S21}$$

Note that time-translation invariance imposes that Y only depends on the time difference $t-t'$. Similarly to Eq. (S19), the two terms in (S21) can be expressed as a sum over three series of times $\{t_i(m), t_j(m'), t_l(m'')\}$. Only terms where the three times are equal give a true third-order contribution and contribute to $Y(t-t')$,

$$\begin{aligned} Y(t-t') &= \sum_m \int_{-\infty}^{\min(t,t')} dt'' r(m)\, m^3 \left(\exp[-k(t+2t')+3kt''] - \exp[-k(2t+t')+3kt'']\right) \\ &= \left[\frac{1}{3k}\sum_m r(m)\, m^3\right] \left[\, \exp(-k|t-t'|) - \exp(-2k|t-t'|)\,\right] \operatorname{sign}(t-t')\,, \end{aligned} \tag{S22}$$

where $\operatorname{sign}(t) = 1$ if $t > 0$ and -1 otherwise.

In order to compare formulas (S17,S19,S22) to direct simulations, it remains to express the Poisson rates $r(m)$ in term of the fusion kernel of the rate equations and the stationary concentrations of diffusing clusters c_n^*.

For an immobile domain that encounters mobile diffusing clusters, the fusion kernel only depends on the size of the diffusing clusters and one has directly $r(m) = KD_0 m^{-\sigma} c_m^*$.

For the case of a diffusing cluster encountering other diffusing clusters (Eq. (S15)) , the fusion times $t_i(m)$ are given by *inhomogeneous* Poisson rates $r(m, n(t))$. Namely, the rate of diffusive encounters with clusters of size m is time dependent since it not only depends on m but also on the time-evolving size $n(t)$ of the followed cluster. In order to apply the above analysis, we approximate $n(t)$ by its average $\langle n\rangle$ and the time-dependent rates $r(m, n(t))$ by the corresponding time independent ones $r(m, \langle n\rangle)$. We thus take

$$r(m) = KD_0(m^{-\sigma} + \langle n\rangle^{-\sigma})\, c_m^*\,. \tag{S23}$$

The average domain size $\langle n\rangle$ is now determined in a self-consistent way by Eq. (S18),

$$\langle n\rangle = \frac{1}{k}\sum_m m\, KD_0(m^{-\sigma} + \langle n\rangle^{-\sigma})\, c_m^* = \frac{c_0 KD_0}{k}\, \langle n\rangle^{-\sigma} + \frac{KD_0}{k}\sum_m m^{1-\sigma} c_m^*\,. \tag{S24}$$

The curves (dashed grey lines) shown in Fig. 4C and 4D of the main text correspond respectively to Eq. (S19) and (S21) with the rate chosen as described in Eq. (S23) and (S24). We finally note that in the framework of the approximate Eq. (S16), the size $n(t)$

of the domain is a continuous variable instead of a discrete one. This is not a significant difference for large clusters. However, with the dynamical evolution (S16), the size $n(t)$ can become arbitrarily small but it cannot vanish. This allows $n(t)$ to really be a stationary process and time averages to be strictly meaningful. For the discrete process, $n(t)$ has a finite probability per unit time to vanish and the domain eventually disappears. There is only a quasi-stationary distribution [11] and time averages from time 0 to t should be understood as averages on conditioned on clusters that survive until t. The very small probability of cluster disappearance per unit time is not significant for the quantities here considered. It is however an interesting quantity since it determines the survival time of an evolving cluster. We plan to analyze it elsewhere.

---

[1] Abramowitz M, Stegun IA. Handbook of mathematical functions: with formulas, graphs, and mathematical tables. Dover,New York; 1972.

[2] Smoluchowski Mv. Grundriß der Koagulationskinetik kolloider Lösungen. Colloid & Polymer Science. 1917;21(3):98–104.

[3] White WH. On the form of steady-state solutions to the coagulation equations. Journal Of Colloid And Interface Science. 1982;87(1):204–208.

[4] Rácz Z. Aggregation in the presence of sources and sinks: A scaling theory. Physical Review A. 1985;32(2):1129–1133.

[5] Turner MS, Sens P, Socci ND. Nonequilibrium raftlike membrane domains under continuous recycling. Physical Review Letters. 2005;95(16):168301.

[6] Bender CM, Orszag SA. Advanced mathematical methods for scientists and engineers I. Springer Science & Business Media; 1999.

[7] Gillespie DT. Exact stochastic simulation of coupled chemical reactions. The Journal of Physical Chemistry. 1977;81(25):2340–2361.

[8] Vagne Q, Turner MS, Sens P. Sensing Size through Clustering in Non-Equilibrium Membranes and the Control of Membrane-Bound Enzymatic Reactions. PloS one. 2015;10(12):e0143470.

[9] Pomeau Y. Symétrie des fluctuations dans le renversement du temps. Journal de Physique. 1982;43(6):859–867.

[10] Pomeau Y, Le Berre M, Ginibre J. Ultimate statistical physics: fluorescence of a single atom.

Journal of Statistical Mechanics: Theory and Experiment. 2016;2016(10):104002.

[11] Collet P, Martínez S, San Martín J. Quasi-stationary distributions: Markov chains, diffusions and dynamical systems. Springer Science & Business Media; 2012.
